# North-South Asymmetry in the Geographic Location of Auroral Substorms correlated with Ionospheric Effects

**DOI:** 10.1038/s41598-018-35091-2

**Published:** 2018-11-22

**Authors:** Kan Liou, Thomas Sotirelis, Elizabeth J. Mitchell

**Affiliations:** The Johns Hopkins University Applied Physics Laboratory, Laurel, Maryland 20723 USA

## Abstract

Energetic particles of magnetospheric origin constantly strike the Earth’s upper atmosphere in the polar regions, producing optical emissions known as the aurora. The most spectacular auroral displays are associated with recurrent events called magnetospheric substorms (aka auroral substorms). Substorms are initiated in the nightside magnetosphere on closed magnetic field lines. As a consequence, it is generally thought that auroral substorms should occur in both hemispheres on the same field line (i.e., magnetically conjugated). However, such a hypothesis has not been verified statistically. Here, by analyzing 2659 auroral substorms acquired by the Ultraviolet Imager on board the NASA satellite “Polar”, we have discovered surprising evidence that the averaged location for substorm onsets is not conjugate but shows a geographic preference that cannot be easily explained by current substorm theories. In the Northern Hemisphere (NH) the auroral substorms occur most frequently in Churchill, Canada (~90°W) and Khatanga, Siberia (~100°E), up to three times as often as in Iceland (~22°W). In the Southern Hemisphere (SH), substorms occur more frequently over a location in the Antarctic ocean (~120°E), up to ~4 times more than over the Antarctic Continent. Such a large difference in the longitudinal distribution of north and south onset defies the common belief that substorms in the NH and SH should be magnetically conjugated. A further analysis indicates that these substorm events occurred more frequently when more of the ionosphere was dark. These geographic areas also coincide with regions where the Earth’s magnetic field is largest. These facts suggest that auroral substorms occur more frequently, and perhaps more intensely, when the ionospheric conductivity is lower. With much of the magnetotail energy coming from the solar wind through merging of the interplanetary and Earth’s magnetic field, it is generally thought that the occurrence of substorms is externally controlled by the solar wind and plasma instability in the magnetotail. The present study results provide a strong argument that the ionosphere plays a more active role in the occurrence of substorms.

## Introduction

Magnetospheric substorms (aka auroral substorms or simply substorms) are one of the major disturbances in the near-Earth space and are not unique to the Earth^1^. Since the framework for auroral substorms was laid out more than half a centry ago^2^, the morphology of an auroral substorm has been studied intensively using ground and space-based imagers. An important aspect of auroral substorms is that they are nighttime phenomena and occur most frequently 1–2 hours before local midnight^3^. Substorms involve reconfiguration of the magnetotail field and disruption of the cross-tail current^4^. The cross-tail current disruption forms a new current system called the substorm current wedge (SCW)^5,6^, which diverts the disrupted tail current into the ionosphere through field-aligned currents. An important implication of these observations is that substorms are initiated in the magnetosphere on closed magnetic field at ~10 Earth radii (R_E_) from the Earth. Coordinated observations suggest that the onset and its subsequent auroral expansion map to just a few R_E_ tailward of the geosynchronous orbits^7^, where reconfiguration of the magnetotail field from a stretch to a dipolar field takes place. Consequently, it is expected that auroral substorms should occur magnetically conjugated in both hemispheres.

A half-century old, vexing question in space physics is the triggering of substorms. A number of theories have been proposed to explain the physical mechanism that causes the onset of magnetic field dipolarization and the formation of substorm current wedge. Most of the substorm theories are narrowly focused and cannot explain the whole aspect of the substorm phenomenon. The theories can be summarized into three categories. First, substorms are externally triggered by discontinuities in the solar wind, in particular the interplanetary magnetic field. Some substorms have a good temporal coincidence with an increase in the north-south (*B*_*z*_) component (e.g., a northward turning)^8^. Sudden reductions of the global magnetospheric plasma convection or the electric field in the near-Earth plasma sheet reduces energization and earthward drift of the plasma sheet particles. The resulting dawn-to-dusk gradient in proton drift speed causes an azimuthally localized pressure minimum in the near-Earth plasma sheet and development of field-aligned current^9^. The second category of theories is associated with magnetospheric local effects. Some research suggests that substorms are triggered by magnetic field reconnection in the midtail region [e.g.^10,11^], which is also known as the near-Earth neutral line (NENL) model, or plasma instabilities, such as the cross-tail current instability, in the near-Earth tail resulting in cross-tail current disruption^12^ (a.k.a the CD model). The NENL model suggests a mechanism of reconnection in the near-Earth magnetotail at ~25 R_E_, where the reversal of plasma flows were typically observed by Geotail^13^. The substorm current wedge may be initiated by braking of earthward plasma flows, launched by the reconnection, and pileup of magnetic fluxes in the inner magnetosphere (6–12 R_E_)^11^. On the other hand, the CD model suggests that a cross-tail current-driven plasma in the near-Earth plasma sheet leads to the current disruption and subsequently trigger reconnection in the mid-tail region via emitting a rarefaction wave^12^. Although these two mechanisms disagree with each other, both accept the possibility of external trigger. The third category of theories emphasizes the importance of the ionosphere by considering the magnetosphere and ionosphere as a coupled system^14^. A non-specified, enhanced localized electric field in the near-Earth tail launches an Alfvén wave into the ionosphere. Bouncing of the Alfvén wave between the high-conducting ionosphere and the current sheet in the tail establishes a connection between the magnetosphere and ionosphere. Field-aligned currents associated with the Alfvén wave divert the cross-tail current into the ionosphere forming the substorm current wedge.

All of these substorm theories more or less assume that the occurrence of substorms is controlled by the magnetosphere or its interaction with the solar wind^15,16^. Although the third category of theories takes into account the coupling of the magnetosphere and ionosphere, the ionosphere is considered passively in the coupling. Little attention has been paid to the potentially active role the ionosphere may play in the substorm development. This is in spite of the fact that theories exist to explain why the aurora is more intense for lower ionospheric conductivity^17,18^ as well as mounting evidence suggesting a relationship between solar illumination and the occurrence frequency of auroral acceleration events, which produce discrete arcs^19^ or auroral intensity in general^20,21^. Because discrete auroras are prominent features of auroral substorms, it is reasonable to suspect solar illumination can influence the occurrence of auroral substorms. The observational evidence may imply that substorm onset should occur most frequently under a dark sky. Indeed, past work already shows that substorms tend to occur about one hour before local midnight. However, there is no evidence that links substorm onset occurrence/location with the solar illumination. The objective of this study is to test this theory.

## Data and Analysis

In this study, we analyze 2659 auroral substorm onsets (aka auroral breakups), with 2003 from the Northern Hemisphere and 536 from the Southern Hemisphere, identified with global far ultraviolet auroral images acquired by Ultraviolet Imager (UVI)^22^ on-board the NASA’s Polar spacecraft in the manner described by^3^]. UVI is a 2-D, snapshot (~37 *s*) type of camera. The optical sensor has a circular field of view, with filters allowing transmission of two oxygen lines (135.6 and 130.4 nm) and two molecular nitrogen Lyman-Birge-Hopfield bands (~150 ± 10 and ~170 ± 10 nm). Under typical conditions, Polar UVI was capable of imaging the entire auroral zone near its apogee above the pole. Polar was launched on February 24, 1996 and was decommissioned in April, 2008. Because the Polar orbit drifted steadily from the North Pole to lower latitudes, after ~1999 only partial oval images could be acquired. To compensate for this orbital effect, the observed substorm onsets that will be used in the following analysis have been normalized to the total imaging area (see Supplementary Information) available for the onset identification.

**Figure 1:**
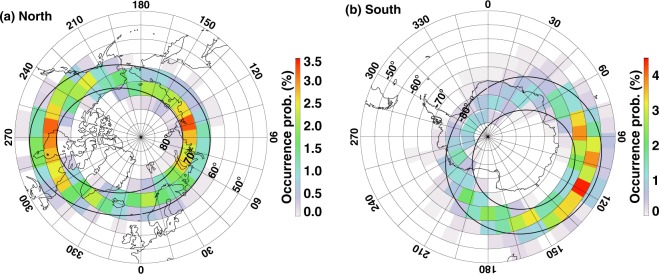
Azimuthal equidistant projection maps of auroral substorm onset occurrence frequency for the (**a**) Northern Hemisphere (NH) and (**b**) Southern Hemisphere (SH). The occurrence frequency is derived from 2659 auroral substorm onsets and averaged in equal-area bins (~5° in latitude). Contours of geographic latitudes are drawn every 5° starting from 45° (−45° in the SH) and longitudes are drawn every 15°. Most of the auroral substorm onsets are initiated between 60° and 75° (−60° and −75° in SH) magnetic latitudes, which are plotted in black contours. Continents are overlapped in black. In NH, the peak onset frequency is ~3.5% and is located west of Hudson Bay, center around Churchill, Canada, and Khatanga, Siberia. In SH the peak onset frequency is ~4.5% and is located in the Antarctic ocean between the Australia and the Antarctic continents.

Figure 1(a,b) show the occurrence frequency of substorm onset in equal-area bins with 5° in latitude and equal length in longitude (~3.1 × 10^5^ km^2^) in the Northern and Southern Hemisphere, respectively. Because of the orbital effect, Polar UVI could not operate continuously in one hemisphere and its field-of-view does not always cover the entire oval. The observed number of substorm onsets in each bin is normalized by the total number of images that contain this bin. As shown in Fig. 1, the occurrence of substorm onset is concentrated in approximately a circular band delineated by two black circles. The inner circle represents magnetic latitudes at 75° and the outer circle represents magnetic latitudes at 60°. The black circles are determined by averages of tens of thousands of auroral images using a thresholding technique. The offset of the oval from its geographic pole is due to the offset of the magnetic pole (currently 11 in the Northern Hemisphere and 17 in the Southern Hemisphere). In general, the occurrence frequency is a few percent for both hemispheres. In the Northern Hemisphere, the occurrence of substorm onset reveals a broad but distinct peak in the northern Canada, with the peak (~3.5%) centering around Churchill, Canada (a small town west of the Hudson bay). A secondary peak (~2.5%) is found in Russia from ~80° to 110° in longitude and from 70° to 75° in latitude. Away from these longitudes, the occurrence frequency drops to ~1% in Alaska and in Iceland. In the Southern Hemisphere, substorm onset occurred predominantly in the Atlantic Ocean, with a peak ~5% and centered at ~57.5° in latitude and ~120° in longitude. The occurrence drops to ~1.5% in the Antarctic continent.

**Figure 2:**
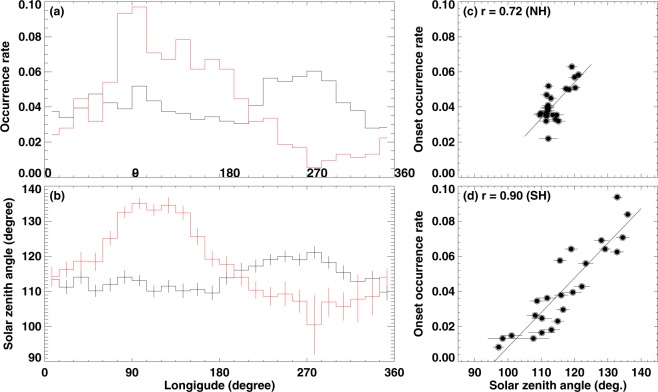
(**a**) Substorm occurrence rates organized by bins of 15° in longitude (hourly time zone) for the Northern (black) and Southern (red) Hemispheres; (**b**) averaged solar zenith angles for the substorm events at different longitudes. The vertical line at each bin represents one-standard deviation of the means. (**c**) and (**d**) scatter plots that show the relationship between the solar zenith angle and substorm onset occurrence rate for NH and SH, respectively. The straight line shows the linear fit of the data points.

In both hemispheres, the peak occurrence frequency of substorm onset occurs in regions of the auroral oval farthest from the geographic pole. These regions receive less sunlight than any other place in the oval for a given day. Because substorms occur every few hours^23^, the long term average presented in this study could have removed any bias. To justify our claim, Fig. 2 shows the averaged solar zenith angle at each hourly sector (15 degrees per hour) of the auroral oval at the substorm onsets. It can be seen that the substorms commenced most frequently at longitude where solar zenith is large (i.e. less sunlight). Moreover, there is a good correlation between the onset occurrence rate and the solar zenith angle (see Fig. 2(c,d)). The Pearson correlation coefficients (r) are r = 0.72 for the NH events and 0.9 for the SH events. Therefore, our result suggests that auroral substorms occur preferentially in a region where the ionosphere is dark.

There are two competing theories that predict intense auroras are more frequent in a dark than a sunlit hemisphere. One invokes the lower plasma density along a geomagnetic field line in winter^24–26^, and the other theory notes that the ionospheric conductivity is much lower under the dark, winter conditions^19,21^. These two factors arise because the bulk of the ionization along the field line or in the auroral ionosphere can arise from the ionization of the background atmosphere by sunlight, and are often indistinguishable. As shown in Fig. 1, there is a pronounced north-south difference in the substorm onset occurrence frequency – a distinct secondary peak in the Northern Hemisphere. In contrast to the major peak, this secondary peak is located in the oval closest to the geographic pole and the ionosphere in this region receives most sunlight in any given day. At a first glance, this particular finding cannot be explained by the two aforementioned theories. Because the magnetic field controls the mobility of the ionospheric charged particles and hence the conductivity. As a result, the background ionospheric conductivity is larger in regions where magnetic field strength is smaller.

**Figure 3:**
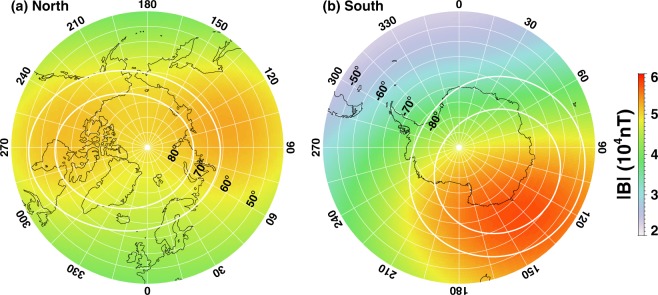
Magnitude of the Earth’s magnetic field at 150 km above the surface of the Earth for (left) Northern Hemisphere and (right) Southern Hemisphere in geographic coordinate system. Contours of geographic latitudes are drawn every 5° starting from 45° and longitudes are drawn every 15°. Areas between the two thick white circles (61.5° and 70.3° magnetic latitude in NH and −63.4° and −72° magnetic latitude in SH) represent the auroral oval. The magnetic field strength is based on the International Geomagnetic Reference Field (IGRF-11) released by the International association of Geomagnetism and Aeronomy (IAGA) for the epoch year 2005.

Figure 3(a,b) show the Earth’s magnetic field intensity |B| at a 150-km altitude based on the International Geomagnetic Reference Field model. It is shown that there are two intensity peaks in |B| in the northern hemisphere: one in Canada and one in Russia. The locations of the peaks approximately coincide with the locations of the primary and secondary onset occurrence frequency peaks. On the other hand, there is only one magnetic field intensity peak in the Southern Hemisphere, roughly coinciding with the onset occurrence frequency peak.

## Discussion

The analysis of 2659 auroral substorm onsets (2003 in the norther and 656 in the southern hemisphere) indicates that substorm onset occurs preferentially in a dark than a sunlit hemisphere. Such a result is consistent with some previous results^19^ which can be explained by the ionospheric feedback mechanism^17,18,27^. Although onset is not the same as the auroral arc, most intense and spectacular auroral arcs are produced at and following the onset. In addition, the typical duration of the substorm expansion phase is no more than 30 min^28^, and the condition of sunlight does not change significantly during such a time period.

If substorms occur randomly or are controlled by external forces, one would expect that the chance for an onset to occur should be the same for the entire oval without any preference. However, our analysis also indicates that the location where substorm onset takes place is not evenly distributed in both northern and southern auroral ovals. The most probable onset location is in the very distant part of the oval from the geographic pole, with an exception that a secondary peak appears in the northern hemispheric oval (~105 °E). This result provides a strong evidence that substorm onset is not controlled entirely by the magnetosphere.

As shown in Figs 1 and 2, there is a pronounced north-south difference in the substorm onset occurrence frequency – two substorm onset occurrence peaks in the Northern Hemisphere and just one in the Southern Hemisphere. Interestingly, there are two peaks in |***B***| in the northern hemisphere and a single peak in |***B***| in the Southern Hemisphere. These peak locations roughly coincide with the peak locations of onset occurrence frequency. Because the mobility of electrons is inversely proportional to |***B***|, a larger |***B***| implies a smaller conductivity. Therefore, the present result supports the ionospheric conductivity feedback mechanism but not the one associated with the ionospheric scale height. Furthermore, a larger |***B***| presence in Southern than Northern Hemisphere results in a smaller conductivity in Southern than Northern Hemisphere. Our result shown in Fig. 2 also indicates that a higher substorm occurrence frequency in the Southern Hemisphere than in the Northern Hemisphere, further supporting the ionospheric feedback hypothesis. The combined effect of the solar illumination and the Earth’s magnetic field strength is probably the main reason for the low (high) correlation shown in Fig. 1 for the northern (southern) hemisphere.

Another possible explanation of this geomagnetic field dependence of substorm onset occurrence is the convergence of the geomagnetic field. A unit area in the ionosphere where the magnetic field is larger maps to a larger area in the magnetosphere. To address this issue, the substorm onset occurrence rate is divided by the normalized magnetic field intensity (e.g., the magnetic field at grid center divided by the hemispheric maximum) and found that there is no significant difference from that shown in Fig. 2 (see Fig. S2 in Supplementary Information). This is because the longitudinal variations in the magnetic field intensity are smaller than the longitudinal variations in the substorm onset occurrence rate, and therefore cannot account for the observed longitudinal variations in occurrence rate.

It is well-known that auroral substorms are associated with a sudden intensification of the westward ionospheric current (aka westward electrojet)^29^. The magnetosphere clearly is the driver that provides currents into the ionosphere and drives the system^6^. Because the divergence of the Earth’s magnetic field, the area in the ionosphere that connects to the magnetic field goes with |***B***|^−2^. For a given magnetospheric source total current, the current density will become larger for larger value of the magnetic field. The larger the current density is, the brighter the aurora is. To be able to be counted as a substorm, the aurora has to be bright enough. So the finding that a larger substorm onset occurrence at a larger magnetic field region may be associated with the detectability of the imager. If this explanation is correct, it would predict the lowest occurrence rate at ~30° longitude in the Southern Hemisphere where |***B***| is weakest along the oval. However, according to Fig. 2, the smallest substorm occurrence rate occurs at ~270° longitude. Therefore, we may rule out this effect, at least as a major contributor of the substorm north-south asymmetry.

Finally, the azimuthal location of auroral substorm onset depends on the IMF *B*_*y*_ -component^30,31^, dipole tilt^30^, and clock angle^32^. A large north-south displacement in the onset location can occur for a large component of IMF *B*_*y*_-component, dipole tilt, and/or clock angle. In this study, we did not consider such effects. However, these effects are expected to smear the onset location azimuthally by no more than ~1–2 hour in local time and will not likely to be the cause of the solar zenith angle dependence shown in Fig. 2.

## Conclusions

In this study, we examine the location of substorm onset in response to the solar EUV illumination by analyzing a larger number of substorm onsets acquired from Polar UVI from both hemispheres. It is found, surprisingly, that substorm onset is far from north-south symmetric, as predicted by current understanding. Instead it is found that (1) substorm onset is more likely to initiate in a dark than a sunlit oval and (2) there is a geophysical preference in the occurrence rate of the substorm onset. It is also found that the preferred locations of substorm onsets coincide with the local peak of the Earth’s magnetic field (or a minimum in the ionospheric conductivity) and are consistent with the ionospheric feedback mechanism. While the driver of substorms is likely in the magnetosphere, the present work suggests that a small ionospheric conductivity favors the initiation of substorm.

## Electronic supplementary material

Supplement Information
