# Supplementary material for: North-South Asymmetry in the Geographic Location of Auroral Substorms correlated with Ionospheric Effects
Source: Sci Rep. 2018 Nov 22;8:17230. doi: 10.1038/s41598-018-35091-2 (PMC6250675; doi:10.1038/s41598-018-35091-2)
Supplement: Supplementary file 1 — Supplement Information [file 41598_2018_35091_MOESM1_ESM.doc]

**Supplementary Information**

**North-south asymmetry in the geographic location of auroral substorms correlated with ionospheric effects**

Kan Liou^1^, Thomas Sotirelis^1^, and Elizabeth J. Mitchell^1^

*1The Johns Hopkins University Applied Physics Laboratory, Laureo, Maryland 20723, USA*

| 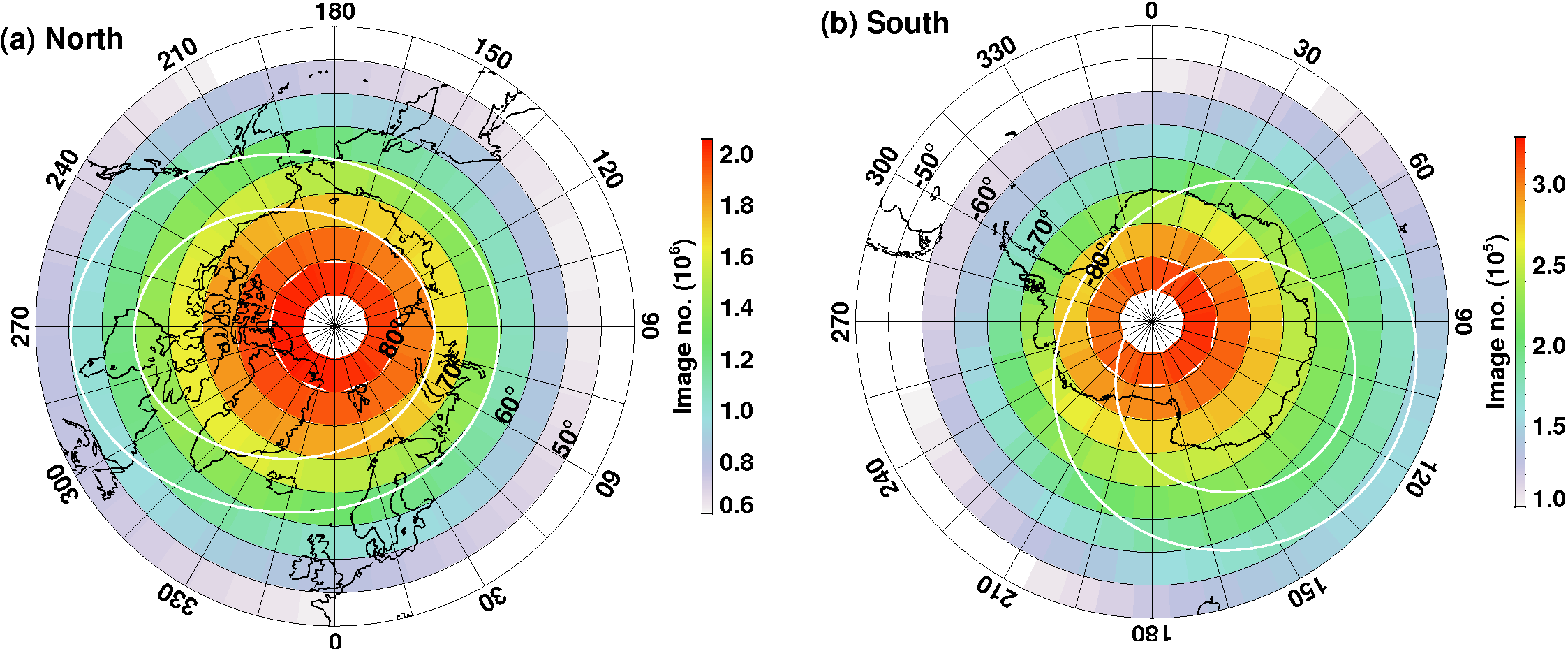 |
| --- |
| **Figure S 1: Distribution of image coverage.** Number of times (in millions) that a geographic region is imaged by Polar UVI in the (a) Northern Hemisphere and (b) Southern Hemisphere. In each panel, the area encased by the two white contours is the the statistical auroral oval. The inner and outer contours represent 75° and 60° magnetic latitude, respectively. |

| 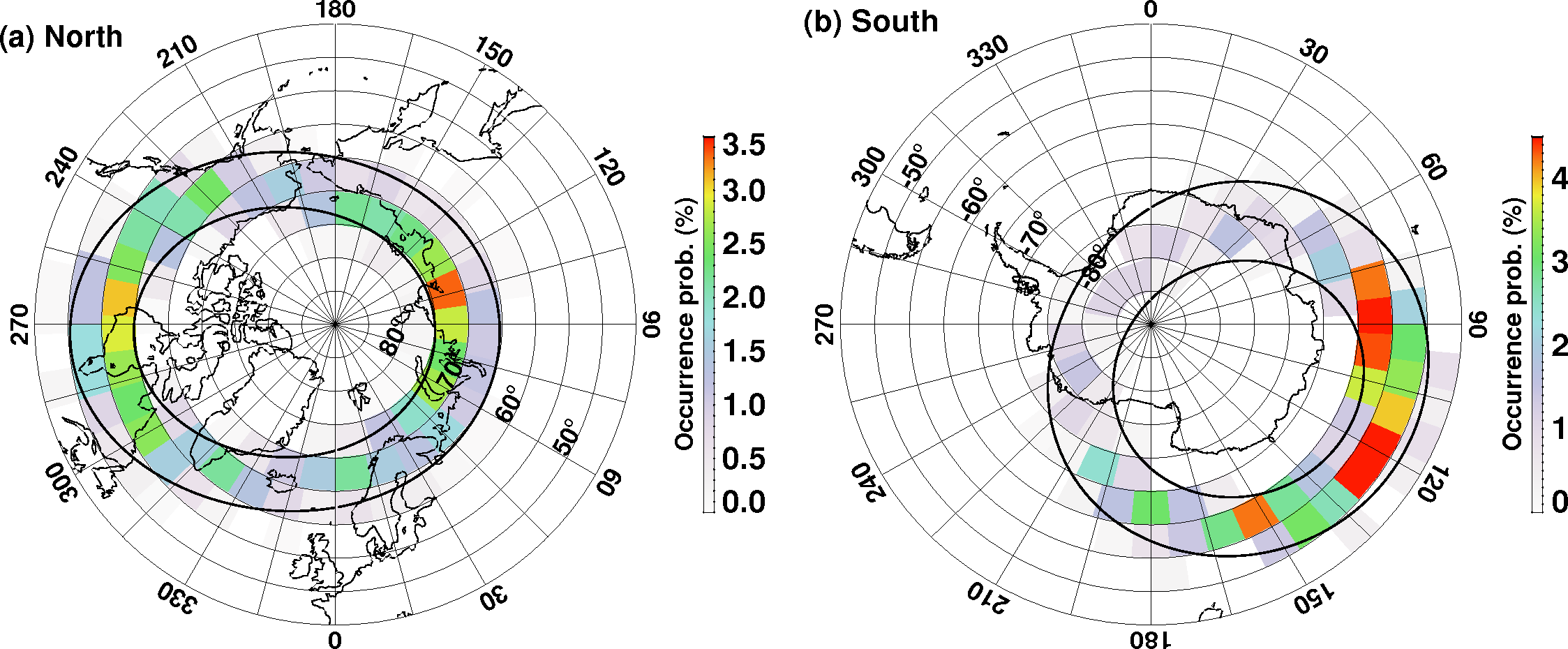 |
| --- |
| **Figure S 2: Magnetic field normalized substorm onset occurrence rate.** Similar to Figure 1 except this figure takes the substorm onset occurrence rate and divide it by the normalized intensity of geomagnetic field, i.e., normalized by it maximum value. The statistical auroral oval is confined between the two black contours in each panel. |
